# Serum BAFF and APRIL Levels, T-Lymphocyte Subsets, and Immunoglobulins after B-Cell Depletion Using the Monoclonal Anti-CD20 Antibody Rituximab in Myalgic Encephalopathy/Chronic Fatigue Syndrome

**DOI:** 10.1371/journal.pone.0161226

**Published:** 2016-08-18

**Authors:** Sigrid Lunde, Einar K. Kristoffersen, Dipak Sapkota, Kristin Risa, Olav Dahl, Ove Bruland, Olav Mella, Øystein Fluge

**Affiliations:** 1 Department of Oncology and Medical Physics, Haukeland University Hospital, Bergen, Norway; 2 Department of Immunology and Transfusion Medicine, Haukeland University Hospital, Bergen, Norway; 3 Department of Clinical Science, University of Bergen, Haukeland University Hospital, Bergen, Norway; 4 Department of Clinical Medicine, University of Bergen, Haukeland University Hospital, Bergen, Norway; 5 Department of Medical Genetics and Molecular Medicine, Haukeland University Hospital, Bergen, Norway; Universidad de Sevilla, SPAIN

## Abstract

Myalgic Encephalopathy/Chronic Fatigue Syndrome (ME/CFS) is a disease of unknown etiology. We have previously suggested clinical benefit from B-cell depletion using the monoclonal anti-CD20 antibody rituximab in a randomized and placebo-controlled study. Prolonged responses were then demonstrated in an open-label phase-II study with maintenance rituximab treatment. Using blood samples from patients in the previous two clinical trials, we investigated quantitative changes in T-lymphocyte subsets, in immunoglobulins, and in serum levels of two B-cell regulating cytokines during follow-up. B-lymphocyte activating factor of the tumor necrosis family (BAFF) in baseline serum samples was elevated in 70 ME/CFS patients as compared to 56 healthy controls (p = 0.011). There were no significant differences in baseline serum BAFF levels between patients with mild, moderate, or severe ME/CFS, or between responders and non-responders to rituximab. A proliferation-inducing ligand (APRIL) serum levels were not significantly different in ME/CFS patients compared to healthy controls at baseline, and no changes in serum levels were seen during follow-up. Immunophenotyping of peripheral blood T-lymphocyte subsets and T-cell activation markers at multiple time points during follow-up showed no significant differences over time, between rituximab and placebo groups, or between responders and non-responders to rituximab. Baseline serum IgG levels were significantly lower in patients with subsequent response after rituximab therapy compared to non-responders (p = 0.03). In the maintenance study, slight but significant reductions in mean serum immunoglobulin levels were observed at 24 months compared to baseline; IgG 10.6–9.5 g/L, IgA 1.8–1.5 g/L, and IgM 0.97–0.70 g/L. Although no functional assays were performed, the lack of significant associations of T- and NK-cell subset numbers with B-cell depletion, as well as the lack of associations to clinical responses, suggest that B-cell regulatory effects on T-cell or NK-cell subsets are not the main mechanisms for the observed improvements in ME/CFS symptoms observed in the two previous trials. The modest increase in serum BAFF levels at baseline may indicate an activated B-lymphocyte system in a subgroup of ME/CFS patients.

## Introduction

Myalgic Encephalopathy/Chronic Fatigue Syndrome (ME/CFS) is a disease of unknown etiology affecting approximately 0.1–0.2% of the population [1]. ME/CFS has a genetic predisposition [2], affects women 3–4 times more often than men, and is often triggered by infections [3]. The disease is characterized by fatigue not alleviated by rest, post-exertional malaise, cognitive disturbances, muscle and joint pain, headache, sleep problems, hypersensitivity to sensory stimuli, and frequently a range of other symptoms related to the immune and autonomic systems [4]. There is a growing interest in the role of the immune system in the etiology of ME/CFS. Abnormalities of cytokine levels and lymphocyte subsets in peripheral blood have been observed [5–9].

In a pilot case series [10], followed by a phase II randomized and placebo-controlled trial (KTS-1-2008) [11], we found that B-cell depletion using the monoclonal anti-CD20 antibody rituximab was associated with clinical improvement in a subgroup of ME/CFS patients. In a subsequent open-label phase II trial (KTS-2-2010) using rituximab induction and maintenance to prolong the B-cell depletion period, prolonged clinical response durations were observed [12].

Thus, based on observations from those two clinical trials, with a lag time of minimum two and up to eight months from initial B-cell depletion until start of clinical responses, we hypothesized that ME/CFS in a subgroup of patients could be an immunological disease, possibly involving B-cells and antibodies (long half-life) for the symptom maintenance. Further arguments supporting this hypothesis are a high occurrence of autoimmunity among first-degree relatives of patients in the previous two clinical studies [11,12], a modest but significantly increased risk of B-cell lymphomas among elderly ME/CFS patients suggesting a chronically activated B-cell system [13], a female preponderance of ME/CFS as shown in established autoimmune diseases, and the often abrupt start of ME/CFS after infections. Also, emerging data from the partly overlapping syndrome Postural Orthostatic Tachycardia Syndrome (POTS) has suggested an immunological disease mechanism. POTS is a frequent finding among ME/CFS patients. One study of 59 patients with ME/CFS showed POTS in 27% (defined as a heart rate increase >30, or heart rate 120) upon standing 10 min [14], another study have shown POTS frequency in 13% of ME/CFS patients [15]. An autoimmune pathogenesis for POTS has been suggested, demonstrating functional autoantibodies to adrenergic receptors with specific binding of autoantibody-positive POTS sera to β1-adrenergic receptor, β2-adrenergic receptor, and α1-adrenergic receptor in transfected cells [16]. In a recent editorial [17], POTS was suggested to belong to the group of autoimmune diseases. Interestingly, elevated serum levels of some of the same autoantibodies have recently been described also in ME/CFS [18] showing that 29.5% of patients with CFS had elevated antibodies against one or more of muscarinic acetylcholine receptors and beta-adrenergic receptors.

The mechanisms by which B-lymphocyte depletion results in clinical responses in a subgroup of ME/CFS patients are currently unknown. In the present study, we used blood and serum samples from patients included in the two mentioned clinical studies KTS-1-2008 and KTS-2-2010, and from healthy controls, to investigate possible associations between the parameters investigated and clinical data such as ME/CFS patients vs healthy controls, mild versus moderate versus severe ME/CFS, and response versus no response after B-cell depletion therapy. The clinical data including inclusion criteria, primary and secondary endpoints, clinical assessments, response characterizations during follow-up (for 12 months in KTS-1-2008, and 36 months in KTS-2-2010) have been described [11,12] and are not repeated in this manuscript. Therefore, we refer only to clinical response status (clinical response versus no response) and the ME/CFS severities (mild, moderate, severe).

Because previous experiences from the clinical trials using rituximab therapy suggested that B-cells might be implicated in the pathogenesis for a subgroup of patients, we focused on the B-cell survival factors BAFF and APRIL.

BAFF is a cytokine produced mainly by innate immune cells, but also by stromal cells and epithelial cells in the gut and airways amongst others. The transmembrane protein can be cleaved to a soluble form and bind to its receptors BAFF receptor (BAFF-R), transmembrane activator-calcium modulator and cyclophilin ligand interactor (TACI), and B cell maturation antigen (BCMA), found on different types of B-cells, and to some extent on other immune cells such as T-cells [19,20].

APRIL is a homologue of BAFF, which also signals through the TACI and BCMA receptors. These cytokines have largely overlapping functions in B-cell maturation, survival and homeostasis, immunoglobulin class switching, maintenance of germinal centres, T-dependent and -independent antibody responses, and in T-cell activation and co-stimulation [21]. High BAFF levels during B-cell maturation promote survival at the expense of B-cell tolerance and may thus lead to the survival of low affinity self-reactive B-cells. Increased BAFF serum levels have been reported in autoimmune diseases, allergic asthma and lymphoid malignancy [19,22].

The cytokines BAFF and APRIL, acting as proliferation factors for B-lymphocytes regulating maturation and cell numbers [23], were analysed through follow-up. The baseline BAFF and APRIL cytokine levels in ME/CFS patients were also compared to healthy controls.

As B-cells are both regulated by, and are regulators of, other lymphocyte subsets, including NK-cells and T-cells, we immunophenotyped lymphocyte subsets in peripheral blood during follow-up in the previous two clinical trials. Changes in serum immunoglobulin levels following B-cell depletion, from baseline to 24 months follow-up, were investigated in patients participating in the KTS-2-1010 study with rituximab maintenance treatment, to assess the extent of reductions and thus possible risks of developing hypogammaglobulinemia.

## Materials and Methods

### Patient Inclusion and Treatment Protocols

A total of 70 ME/CFS patients were available for baseline BAFF analyses, including 38 patients participating in the previous rituximab clinical trials who had repeated blood samples through follow-up. For the remaining 32 other ME/CFS patients, only baseline blood samples were available. The KTS-1-2008 trial (NCT00848692) included 30 ME/CFS patients, 15 in the rituximab group and 15 in the placebo group [11]. In the open-label phase-II trial KTS-2-2010 (NCT01156909) exploring rituximab induction followed by maintenance, 29 patients participated, including two pilot patients, 9 patients from the placebo group in KTS-1-2008, and 9 patients given rituximab in the KTS-1-2008 study having either clinical response and subsequent relapse (n = 6), or no response (n = 3) [12]. In both KTS-1-2008 and KTS-2-2010, patients were included according to Fukuda criteria [24], however, all but two patients also met the Canadian criteria for ME/CFS [4]. Summarized, approximately 60% of rituximab-treated patients experienced clinical response in these two clinical trials, and in the KTS-2-2010 study maintenance rituximab therapy with prolonged B-cell depletion period was associated with prolonged response durations. Among 18 responders in the KTS-2-2010 study, mean response duration was 97 weeks within the 156 weeks study period, and with 11 patients still in remission at end of follow-up [12]. The 32 other ME/CFS patients included for baseline blood samples all fulfilled Canadian criteria. The Regional Committee for Medical and Health Research Ethics and the National Medicines Agency in Norway approved the clinical trials. Written consent was obtained from all patients.

### Treatment Regimes

Patients in the active treatment arm (n = 15) in KTS-1-2008 received rituximab, two infusions administered two weeks apart (each 500 mg/m^2^, max 1000 mg), whereas the placebo arm (n = 15) received equal volumes of saline [11]. The patients were assessed at 2, 4, 6, 8, 10 and 12 months without further drug intervention during follow-up. In KTS-2-2010 a total of 28 (out of 29 included) patients received rituximab induction with two infusions two weeks apart. Thereafter additional maintenance rituximab infusions were given after 3, 6, 10 and 15 months (all 500 mg/m^2^, max 1000 mg) [12]. Treatment could be discontinued if no signs of clinical response were observed after 10 months follow-up, thus omitting the planned 10 and 15-months rituximab infusions. One patient in KTS-2-2010 had an allergic reaction at the end of the first rituximab infusion and did not receive further infusions. According to an approved amendment in the KTS-2-2010 trial, 7 patients in moderate response after 12 months follow-up with gradual but on-going symptom improvement (among 18 responders to rituximab according to predefined criteria) received additional rituximab treatment beyond 15 months follow-up with a range of 2–5 extra infusions. The inclusion criteria, endpoints, response characterizations and follow-up data have been published [11,12]. In the present study, we have analysed biobank samples taken up to and including the 24 months follow-up where available.

### Peripheral Blood Collection

Baseline blood samples from 70 ME/CFS patients and 56 healthy individuals were collected by venous puncture. The patients included in the two clinical trials, KTS-1-2008 and KTS-2-2010, had serial blood samples drawn and analysed for alterations over time following B-cell depletion. When rituximab or placebo was administered, samples were drawn prior to infusions. Serum and/or plasma samples were stored at -80°C prior to any analyses. EDTA anticoagulated blood samples were used for immunophenotyping by flowcytometry to determine lymphocyte subsets.

### Serum Cytokine and Immunoglobulin Levels

Serum levels of BAFF and APRIL cytokines were determined at baseline and at specific time points during follow-up using BAFF (R&D Systems, UK) and APRIL (Invitrogen/Life Technologies, UK) ELISA kits following the manufacturers’ instructions. Samples were run in duplicates. For the BAFF and APRIL ELISA analyses, sera from 56 or 22 healthy blood donors, respectively, were included for comparison. The plates were read using a FLUOstar Omega plate reader (BMG Labtech, Germany). Serum BAFF and APRIL levels were quantified using the in-kit standard curves for the respective cytokines. To adjust for technical variations in measurements of absolute BAFF concentrations between plates, all samples were calibrated using a reference plate containing several samples from all other plates.

Serum immunoglobulin levels (IgG, IgA, and IgM) were measured for all time points up to 24 months after intervention for patients included in KTS-2-2010 trial using a Siemens BN ProSpec Nephelometer (Erlangen, Germany).

### Immunophenotyping

For patients included in the KTS-1-2008 and KTS-2-2010 trials, lymphocyte sub-populations (T- and NK-cells) were analysed in EDTA anticoagulated blood samples, before treatment and during follow-up visits. Immunophenotyping was performed using the BD Multitest 6-color TBNK kit with BD Trucount Tubes for relative and absolute concentration determination (CD16/56 PE, CD8 APC-Cy7, CD3 FITC, CD19 APC, CD45 PerCP-Cy5.5, CD4PE-Cy7) (BD Biosciences, Cupertino, CA, USA). The samples were prepared according to the manufacturer’s instructions and immediately analysed on a BD Canto II flow cytometer (BD Biosciences). T-cells were identified as CD3+, with T helper cells being CD3+ and CD4+, and cytotoxic T-cells being CD3+ and CD8+. NK-cells were identified as CD56+ and CD16+, and B-cells identified as CD19+.

In the randomized and double-blinded KTS-1-2008 trial, the immunophenotyping data were not made available to the clinical researchers until after the end of the follow-up period.

Further analyses of T-cell subtypes and T-cell activation markers in EDTA anticoagulated blood samples were performed at all time points in the KTS-1-2008 trial using two panels with eight antibodies. The first panel detected regulatory T-cells and T-cell activation: CD4 FITC (BD Biosciences), CD25 PE (Beckman Coulter, France), CD19 PC5 (Beckman Coulter), CD28 PC7 (eBiosciences, San Diego, USA), HLA-DR APC (BD Biosciences), CD3 APC-Cy7 (eBiosciences), CD127 Pacific Blue (eBiosciences) and CD45 Pacific Orange (Invitrogen/Life Technologies, UK). Regulatory T-cells were defined as CD4+, CD25++ and CD127-.

The second panel for T-cell activation included CD278 (ICOS) FITC (eBiosciences), CD154 PE (Immunotech/Beckman Coulter), CD8 PC5 (Beckman Coulter), CD69 PE-Cy7 (BD Biosciences), CD3 APC (BD Biosciences), CD4 APC-H7 (eBiosciences), HLA-DR Pacific Blue (BioLegend, San Diego, USA), and CD45 Pacific Orange (Invitrogen). All antibodies were titrated for optimal separation and staining intensities of cell populations. Anticoagulated blood were lysed using EasyLyse (DAKO, Denmark,) according to the instructions of the manufacturer, washed by centrifugation in buffered saline, and incubated with the respective antibodies for 15 min at room temperature in the dark. The cells were analysed on a BD Canto II flow cytometer (BD Biosciences), fitted with three lasers to obtain eight fluorochrome parameters in addition to scatter. The data were analyzed using FloJo software (version 7.6.3, Three Star Inc. USA).

### Statistical Analysis

Comparisons of absolute serum BAFF and APRIL levels, between ME/CFS patients and healthy controls, of BAFF levels between responders and nonresponders to rituximab therapy, and of BAFF levels between patients with different disease severity or different disease duration, were performed using unpaired t-tests (equal variances not assumed). For analyses of serum BAFF and APRIL changes over time, and to avoid inter-plate variations, all time-point samples were run on the same plate as their respective baseline samples. The ratios of serum BAFF or APRIL levels at specific time points to baseline (relative values) were used for statistical analyses. Baseline values were set to 1 (reference value) for all patients. The normalized ratios of serum BAFF or APRIL levels at the different time points were then compared to baseline reference values using Repeated measures One-way ANOVA with Dunnett’s test for adjusted p-values for individual comparisons to baseline.

Spearman analyses were used to investigate correlations between serum BAFF levels and other immunological parameters at baseline. Mann-Whitney U-test was used to compare baseline levels of immunoglobulins, between patients with subsequent clinical response versus no response after B-cell depletion therapy in the trials.

For patients in the KTS-2-2010 trial, changes in immunoglobulin levels from baseline to 24-months follow-up were assessed by paired t-tests. Statistical analyses were performed using GraphPad Prism version 6.0 GraphPad Software, CA, USA).

Immunophenotyping data (ratios of CD4+/CD8+, and cell numbers in peripheral blood positive for CD3+, CD4+, CD8+ and CD56/16+, and levels of immunoglobulins IgG, IgA, IgM) were analyzed for changes during the 24 months of follow-up. The data were compared between the responders and non-responders in the KTS-2-2010 study, and between the rituximab- and placebo groups in the KTS-1-2008 study. General Linear Model (GLM) for Repeated Measures with Greenhouse-Geissner correction was used, with estimates for the interaction between time and group, to assess if there were any significant differences between groups during follow-up (rituximab vs placebo, responder vs non-responder). GLM analyses for repeated measures were performed using SPSS software version 22 (IBM-SPSS, Chicago, US). For GLM analyses, missing values during follow-up were replaced by interpolation between preceding and successive values for the actual patient, described in the respective figure legends. At each individual time point during follow-up, the groups were also compared using unpaired t-test.

## Results

### Serum BAFF and APRIL analyses

**Fig 1:**
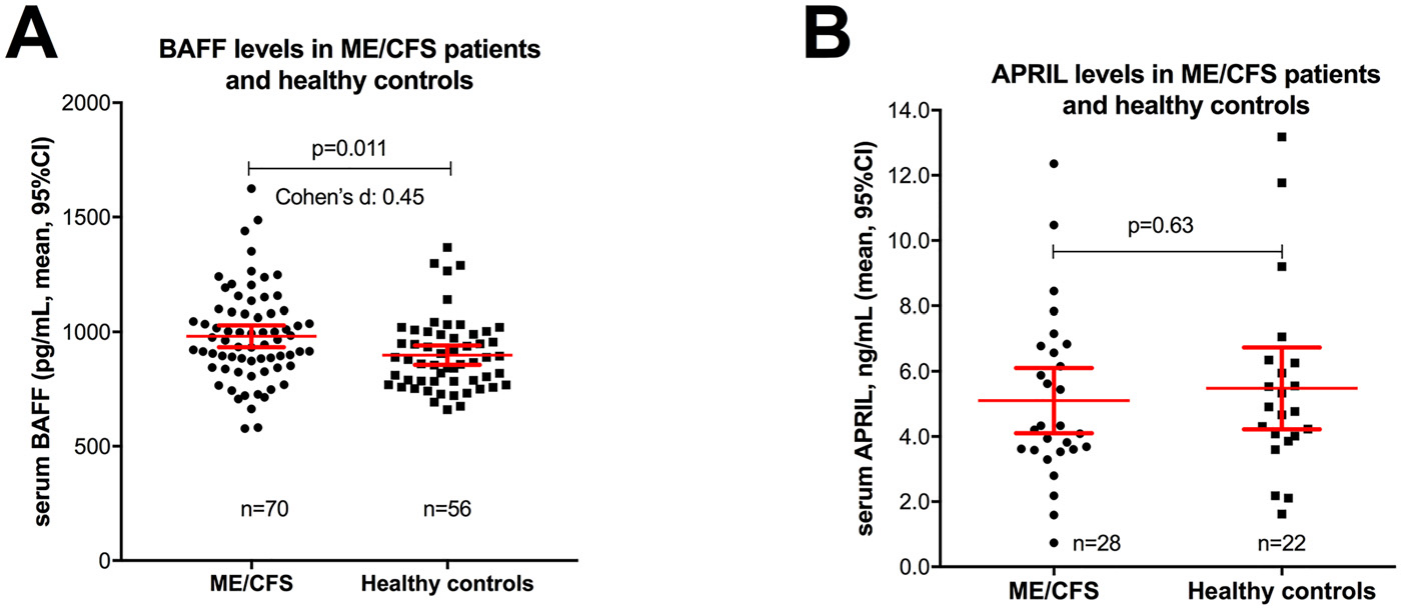
Serum BAFF levels at baseline. Panel A: Baseline serum BAFF levels (pg/mL) in 70 ME/CFS patients and in 56 healthy controls. Serum samples before treatment in the KTS-1-2008 and KTS-2-2010 trials (n = 38), and in addition 32 samples from ME/CFS patients fulfilling Canadian diagnostic criteria, were included. Panel B: Baseline APRIL serum concentrations (ng/mL), in ME/CFS patients included in KTS-1-2008 study (n = 28) and in healthy controls (n = 22). P-values from unpaired t-test (equal variances not assumed). Error bars denote mean and 95% confidence intervals (CI).

In baseline samples from ME/CFS patients (n = 70), the mean serum BAFF level was 981 pg/mL (95%CI 933–1029), while in healthy controls (n = 56) the median serum BAFF level was 899 pg/mL (95% CI 856–940) (p = 0.011) (Fig 1A). The effect size for the difference assessed by Cohen’s d was 0.45. There were no significant differences between the ME/CFS patients and healthy controls in distribution of age (mean 38.3 years in ME/CFS vs 40.5 years in healthy controls, p = 0.31) or of sex (73% women in ME/CFS vs 59% women in healthy controls, p = 0.10).

There were no significant differences in serum BAFF levels at baseline in those with a subsequent clinical response to rituximab compared to those with no response during follow-up. Further, there were no significant differences in the baseline serum BAFF levels between patients with mild, moderate or severe ME/CFS according to the NICE-guidelines [25], or between baseline serum BAFF levels and disease duration categorized as 2–5, 5–10 and >10 years (data not shown).

**Fig 2:**
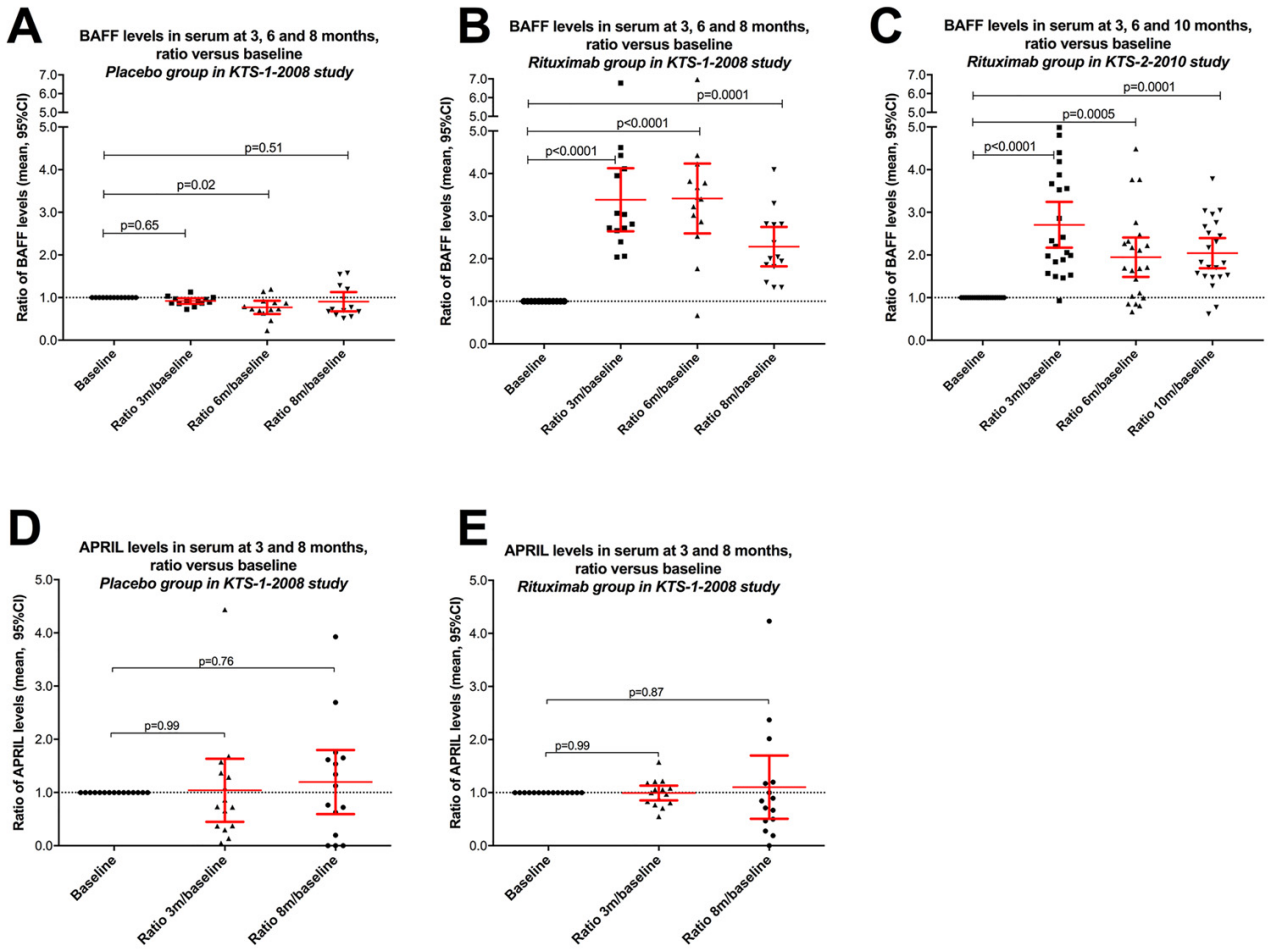
Serum BAFF levels during follow-up in the KTS-1-2008 and KTS-2-2010 trials. Panel A: Serum BAFF levels at baseline and at 3, 6, and 8-months follow-up in the placebo arm of the KTS-1-2008 trial, relative to baseline levels (n = 13). Panel B: Serum BAFF levels at baseline, and at 3, 6, and 8-months follow-up in the rituximab arm of the randomized KTS-1-2008 trial, relative to baseline levels (n = 14). Panel C: Serum BAFF levels at baseline and at 3, 6, and 10-months follow-up in the open-label KTS-2-2010 rituximab maintenance trial, relative to baseline levels (n = 22). Panel D: Serum APRIL levels at baseline, and at 3 and 8-months follow-up in the placebo arm of the KTS-1-2008 trial, relative to baseline levels (n = 12). Panel E: Serum APRIL levels at baseline, and at 3 and 8-months follow-up in the rituximab arm of the KTS-1-2008 trial, relative to baseline levels (n = 13). P-values from Repeated measures One-way ANOVA with Dunnett’s test for adjusted p-values for individual comparisons of the different time points to baseline. Error bars denote mean and 95% CI.

Following intervention in the rituximab group in KTS-1-2008, serum BAFF levels increased significantly from baseline levels, at 3 months (p<0.0001), 6 months (p<0.0001) and at 8 months (p = 0.0001) (Fig 2B). In contrast, and as expected, for patients in the placebo group, no increase in serum BAFF levels occurred during follow-up (p = 0.09 for overall difference by Repeated Measures One-way ANOVA), although the mean serum BAFF level at 6 months was slightly but significantly lower than at baseline (p = 0.018) (Fig 2A). Significant increases in serum BAFF levels, as compared to baseline, were also observed in KTS-2-2010 at 3 months (p<0.0001), 6 months (p = 0.001), and at 10 months (p<0.0001) (Fig 2C). Thus, in both clinical trials, serum BAFF levels were increased following B-cell depletion, as expected.

In contrast to serum BAFF levels, there was no significant difference between the serum APRIL levels in ME/CFS patients from KTS1-2008 (n = 28, baseline samples) and healthy controls (n = 22) (Fig 2D). Furthermore, there were no significant changes in serum APRIL levels during follow-up at 3 or at 8 months, as compared to baseline, either in the rituximab- or placebo groups (Fig 2E).

In ME/CFS patients, baseline serum BAFF levels did not correlate significantly with HLA-DR on T-cells, for HLA-DR (%CD4) r = 0.29, p = 0.18, and for HLA-DR (%CD8) r = 0.26, p = 0.16. There was no significant correlation between BAFF levels and number of CD19+ B-cells at baseline (r = 0.11), although the inverse relation was evident after B-cell depletion (Fig 2). Immunoglobulin levels at baseline were not significantly associated with either number of CD19+ cells or with serum BAFF levels.

### Immunophenotyping of Lymphocyte Subsets

**Fig 3:**
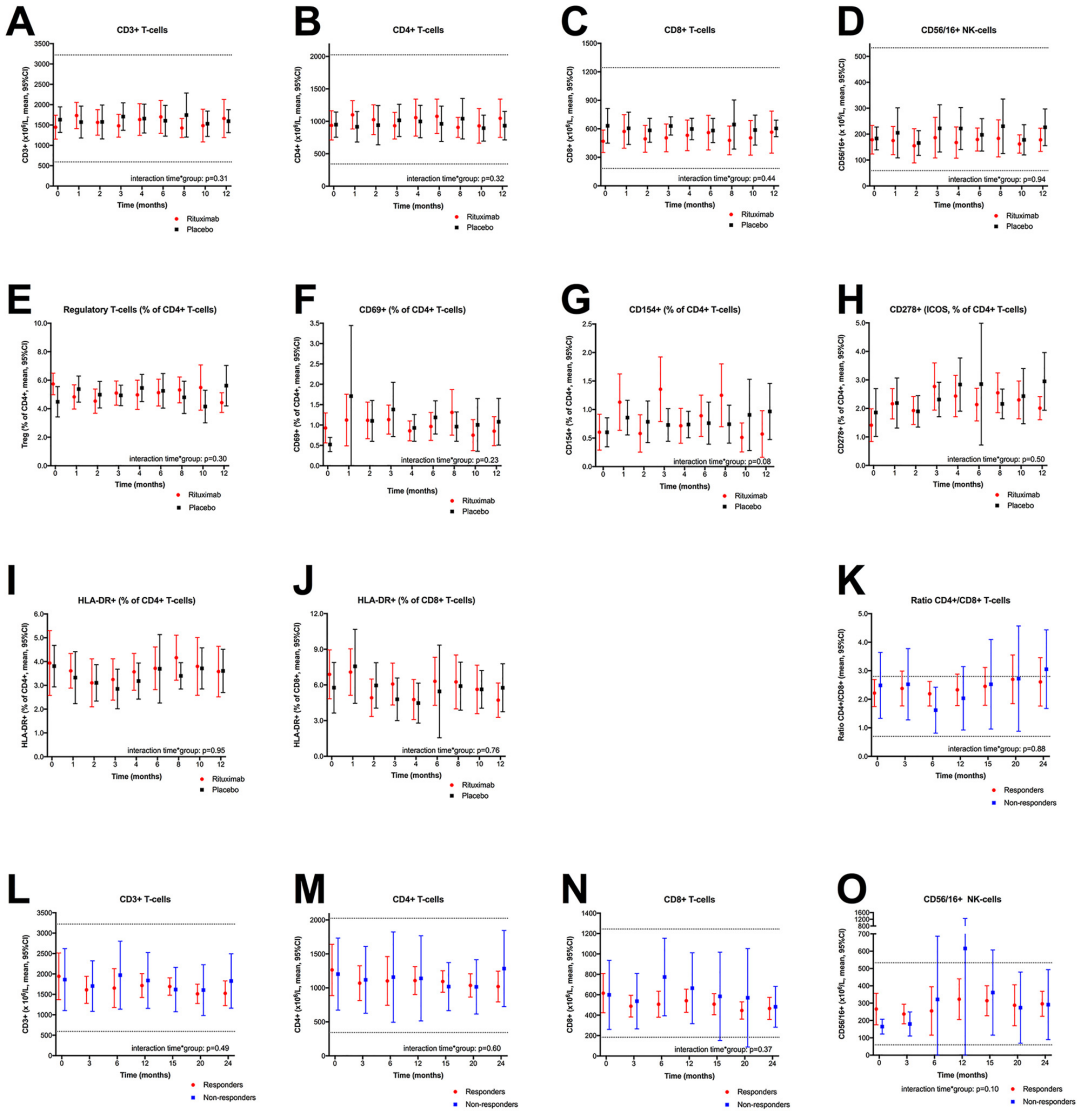
Lymphocyte subsets during follow-up in the KTS-1-2008 and KTS-2-2010 trials. Panels A-D: Lymphocyte subsets among patients included in the randomized KTS-1-2008 study, in the rituximab group (red) and the placebo group (black), determined by flowcytometry and shown as million cells per liter at baseline (0 months), and at 1, 2, 3, 4, 6, 8, 10, and 12 months follow-up (n = 30). Panel A: CD3+ T-cells, panel B: CD4+ T helper cells, panel C: CD8+ cytotoxic T-cells, panel D: CD56+/16+ NK-cells. For 29 patients, data on lymphocyte populations were missing for 16 out of 261 (6.1%) time points. For General linear model (GLM) for repeated measures these were replaced by interpolation between preceding and succeeding values. The missing values were not replaced in the plots. Panels E-J: T-cell activation markers among patients included in the KTS-1-2008 trial, at baseline and through 12-months follow-up, and shown as percentage of the CD4+ T helper cell population, as determined by flowcytometry. Panel E: Regulatory T-cells, panel F: CD69+ cells, panel G: CD154+ cells, panel H: CD278+ (ICOS) cells, panel I: HLA-DR+ cells. Panel J shows the percentage of HLA-DR+ cells among the CD8+ cytotoxic T-cells. For 29 patients, data on T-regulatory cells and T-cell activation parameters were missing for 30 out of 261 (11.5%) time points. For GLM, missing values were replaced by interpolation between the preceding and succeeding analyses (not replaced in the plots). Panels K-O: Lymphocyte subsets among patients in the KTS-2-2010 study receiving rituximab maintenance therapy, determined by flowcytometry and shown as million cells per liter at baseline (0 months), and 3, 6, 12, 15, 20, and 24-months follow-up. Responders to B-cell depletion therapy are shown in red and non-responders in blue. Panel K: Ratio of CD4+/CD8+ T-cells, panel L: CD3+T- cells, panel M: CD4+ T helper cells, panel N: CD8+ cytotoxic T-cells, panel O: CD56/16+ NK-cells. In three patients, data on lymphocyte subpopulations for at least three time points were missing, these three were omitted leaving 23 patients for GLM analyses to assess the interaction between time and response group. In these 23 patients, 15/161 (9.3%) data were missing; these were replaced by interpolation between preceding and succeeding values (not replaced in the plots). Analyses for interaction between time and intervention group, i.e. assessing for difference in course of the variables over time, between the rituximab and placebo groups (panels A-J), or between responders and nonresponders to rituximab maintenance treatment (panels K-O), were performed using GLM for repeated measures. Error bars denote 95% confidence intervals (CI) for the mean values. The dotted lines indicate lower and upper normal reference values as established at Haukeland University Hospital.

Lymphocyte subsets in peripheral blood were determined by flow cytometric analyses, at baseline and at several time points during follow-up. In the KTS-1-2008 study, the variations over time in T- or NK-cell numbers in peripheral blood were not different between rituximab treated and placebo patients (Fig 3A–3D). There were no significant differences in cell counts for any of the CD3+, CD4+, CD8+ or CD56/16+ subset analyses, between the rituximab and placebo groups, at any of the specific time points during follow-up (Fig 3A–3D). Further investigations of the T-cell subsets such as regulatory T-cells (CD25++, CD127-), and several T-cell activation markers (CD69+, CD154+, CD278+, HLA-DR+) shown as the percentages of CD4+ cells in peripheral blood, showed no significant interactions between time and randomization group, or any difference in distribution at specific time points, between the rituximab and placebo groups for any of the assessed T-cell subset parameters (Fig 3E–3J).

Furthermore, immunophenotyping data from peripheral blood for T- and NK-cells (CD3+, CD4+, CD8+ or CD56/16+) of patients included in the open-label phase II trial KTS-2-2010 with rituximab induction followed by maintenance, showed no significant interactions between time and response group (responder versus non-responder), or any significant differences in number of cells between the two groups at specific time points, for any of the variables analysed (Fig 3K–3O).

### Serum Immunoglobulins

Repeated measurements of IgG, IgA and IgM up to 24-months follow-up were performed for patients included in the KTS-2-2010 trial with rituximab maintenance treatment.

**Fig 4:**
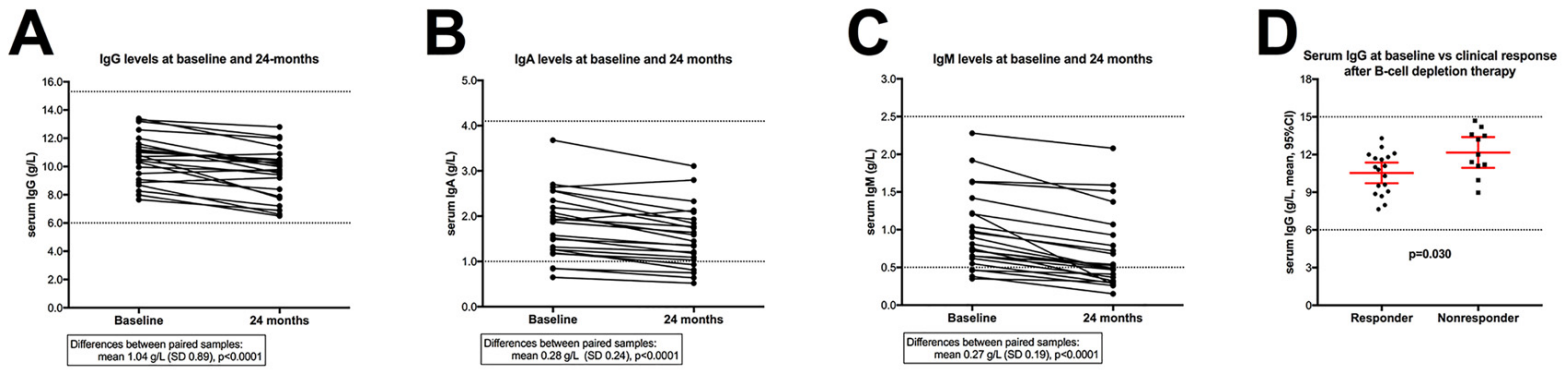
Serum immunoglobulin levels during 24-months follow-up, in KTS-2-2010 trial. Immunoglobulin levels in serum during 24 months follow-up, for patients included in the KTS-2-2010 trial with rituximab maintenance therapy, and shown as grams per liter (g/L). In panels A, B and C are shown serum levels for IgG, IgA, and IgM, respectively, with corresponding values at baseline and at 24-months, for each of 23 patients included in KTS-2-2010. P-values from paired t-tests. Panel D: Baseline levels of serum IgG (g/L), shown for patients with subsequent clinical response to B-cell depletion therapy during follow-up, or patients with no response. P-value from unpaired Mann-Whitney U test. The dotted lines indicate normal reference values as established by Haukeland University Hospital.

From baseline until 24-months follow-up (for 23 patients in the KTS-2-2010 study with immunoglobulin analyses both at baseline and at 24-months follow-up), the IgG levels decreased from a mean 10.6 g/L (SD 1.68) at baseline to a mean 9.54 g/L (SD 1.78) at 24-months (p<0.0001 by paired t-test) representing a 10% relative reduction of IgG level (Fig 4A). The serum IgA levels decreased from a mean 1.81 g/L (SD 0.73) at baseline to a mean 1.53 g/L (SD 0.67) at 24-months follow-up (p<0.0001) representing a 15% relative reduction of a mean IgA level (Fig 4B). The serum IgM levels decreased from mean 0.97 g/L (SD 0.51) at baseline to a mean 0.70 (SD 0.50) at 24-months follow-up (p<0.0001) i.e. 28% relative reduction of mean IgM (Fig 4C).

The baseline levels of serum immunoglobulins, among ME/CFS patients included in the two clinical trials KTS-1-2008 [11] and KTS-2-2010 [12], were analyzed by clinical response versus no response after B-cell depletion therapy. Among 29 patients with available baseline data, there was a significantly higher serum mean IgG level at baseline in 11 non-responders (mean IgG 12.2 g/L, SD 1.7) compared to 18 responders (mean IgG 10.5 g/L, SD1.8), p = 0.030 (Fig 4D). There was no significant difference in percent reduction of total IgG, from baseline to 24 months follow-up. There were no significant differences between patients with clinical response versus no response during follow-up in the trials, for baseline levels of IgA, IgM, BAFF, CD19+, CD4+, or CD8+ (data not shown).

## Discussion

The randomized and placebo-controlled phase-II trial (KTS-1-2008) using the anti-CD20 monoclonal antibody rituximab as a B-lymphocyte depleting intervention for ME/CFS, suggested clinical benefit on ME/CFS symptoms in a subgroup of patients [11]. The open-label phase-II study KTS-2-2010 [12] with rituximab maintenance treatment showed a similar response rate of approximately 60% of patients, and also showed that symptom alleviation could be prolonged with maintenance rituximab treatment. Although B-lymphocyte depletion in peripheral blood occurs within days after rituximab infusion, the responses had a lag time of at least two months and up to eight months, before the patients started to report clinical improvement. This pattern of responses led us to speculate as to whether there is an underlying immune dysfunction in a subgroup of ME/CFS patients, involving B-cells and antibodies, possibly a variant of an autoimmune process compatible with slow washout of antibodies preceding clinical responses. Alternatively, some other slow alteration in immune function governed by circulating B-cells could underlie the disease process. In order to gain further insight into the reason for the efficacy of rituximab in this subgroup of ME/CFS patients, we investigated if there were any changes in selected immune parameters during follow-up between the rituximab- and placebo group in the KTS-1-2008 trial, or between responders and non-responders in the KTS-2-2010 open-label trial with rituximab maintenance treatment.

Baseline serum BAFF level was significantly elevated in ME/CFS patients compared to healthy controls. We found no associations between baseline serum BAFF levels in patients with later clinical response after rituximab therapy versus non-responders, and no differences in baseline BAFF levels in ME/CFS patients of different clinical severity. BAFF acts as an important survival factor during B-cell maturation and functions in the regulation of both innate and adaptive immune responses [26]. Excessive levels of BAFF during maturation causes B-cell hyperplasia and may promote autoimmunity due to enhanced survival of self-reactive B-cells, which are more dependent upon BAFF for survival than non-autoreactive B-cells [27,28]. In addition to emergence of autoreactive cells, high BAFF levels may enhance adaptive immune response through stimulating dendritic cell maturation [26]. Increased BAFF levels have been detected in several autoimmune diseases [26] including rheumatoid arthritis (RA), systemic lupus erythematosus (SLE) [29], primary Sjögren’s syndrome (pSS) [30,31] and myasthenia gravis (MG) [32]. Recently, targeting BAFF has emerged as a therapeutic strategy in SLE [33] and in RA where therapeutic effect was reported [34]. However, increased BAFF level is not a prerequisite for an autoimmune disease [35–37], and studies show that many patients with clinically active SLE and RA have normal BAFF levels. BAFF has also been implicated in additional pathologies, including cancer, allergy (asthma) and infection responses [23].

In baseline samples from ME/CFS patients, there was no significant correlation between BAFF and total CD19+ B-cells, although the inverse relationship was evident after B-cell depletion. Although statistically significant, the difference in baseline serum BAFF levels was relatively modest in magnitude and there was a broad overlap between ME/CFS patients and healthy controls; thus, serum BAFF level is not suitable as a biomarker for the disease.

In both our clinical trials KTS-1-2008 and KTS-2-2010, the BAFF responses to B-cell depletion are in accordance with those previously reported in autoimmune diseases [36–38]. We observed the expected increase in serum BAFF levels following B-cell depletion, with a subsequent gradual decline towards baseline levels. Thus, following rituximab-induced B-cell depletion in ME/CFS-patients, BAFF feedback in the B-cell survival system is as expected.

Contrary to our findings, in patients with SLE baseline elevated serum BAFF levels correlated positively with numbers of CD19+ B-cells [39]. After B-cell depletion therapy, serum BAFF levels were higher at SLE relapse compared to disease flare prior to rituximab treatment. Further, serum BAFF correlated positively with anti-double-stranded DNA antibodies, suggesting a role for BAFF driving SLE disease flares and that sequential B-cell depletion therapy could promote gradually increasing BAFF levels [39]. Transgenic mice overexpressing BAFF may develop a SLE-like disease, showing the importance of the BAFF cytokine in this disease [40]. Whether a similar mechanism could be operative after B-cell depletion therapy in ME/CFS may be investigated after un-blinding of the ongoing randomized, phase-III study in Norway with rituximab maintenance versus placebo (NCT02229942).

APRIL was not significantly influenced either by the ME/CFS disease itself (no difference in serum APRIL levels at baseline from healthy controls) or by the intervention (rituximab versus placebo). The APRIL cytokine plays a role later in B-cell development than BAFF [26]. A differential effect between BAFF and APRIL responses to rituximab has previously been observed [31,36], and may be explained by the fact that while BAFF-R receptor is lost during B-cell depletion, TACI receptor (used by both BAFF and APRIL) remains on both plasma cells (lacking CD20 and therefore not depleted) and on activated B-cells [31].

Abnormalities in cytokine profiles have been reported in ME/CFS [5,6]. However, the pattern of alterations reported varies between studies, among individuals, and over time for individual patients [7]. Other studies reported no consistent abnormalities in cytokine expression in ME/CFS patients as compared to healthy individuals [41]. A study investigating cytokine patterns in peripheral blood from ME/CFS patients [8], revealed abnormalities of both pro- and anti-inflammatory cytokines in the first three years of the disease, which were not present later in the disease course. The same research group also reported a disturbed cytokine pattern in cerebrospinal fluid of ME/CFS patients, compared to healthy controls and multiple sclerosis patients, which could be consistent with an immune activation and a shift towards a Th-2 pattern [42].

The demonstrated increase in serum BAFF levels may be consistent with an activated B-cell system in the ME/CFS patient group. The assumption that a subgroup of ME/CFS patients has a chronically activated B-lymphocyte system is strengthened by a population-based case-control study from the National Cancer Institute (US) [13]. In that study, among almost 1.2 million cancer cases in elderly and 100.000 elderly controls without cancer aged more than 65 years, with a prevalence of ME/CFS 0.5% in both groups, a significant association was found between ME/CFS and B-cell lymphomas [13], and particularly for the low-grade marginal-zone lymphomas known for associations to either chronic infections or autoimmunity. The risk of lymphoma in elderly ME/CFS patients is not of concern to the individual patient, but is yet indicates a biological property of the disease.

An increased risk of B-cell lymphomas has also been reported in several established autoimmune diseases [43], such as SLE [44], and for low-grade marginal-zone lymphomas in pSS [45].

In our present data, we did not compare the flow cytometric data from ME/CFS patients to healthy controls, but rather focused on longitudinal changes during follow-up and associations to intervention. This is a limitation of the present data.

A recent study reported an increased proportion of CD3+, CD8+, and HLA-DR on CD8+ T-cells, and a lower percentage of CD19+ B-cells, among ME/CFS patients compared to healthy controls, but with no associations to ME/CFS severity or disease duration [46]. In both our clinical studies, T-cell numbers (CD3+, CD4+, CD8+, CD56/16+) were in the normal ranges. Serial samples at multiple time-points during follow-up showed no significant differences over time, between patients treated with rituximab or placebo in the KTS-1-2008 study, or between responders and non-responders to B-cell depletion therapy in KTS-2-2010. Also, for patients in the KTS-1-2008 study, we found no significant differences in the T-cell activation parameters from repeated samples during follow-up, between patients in the rituximab- or placebo groups. The T-cell activation parameters assessed were CD278 (Inducible T-cell costimulator, ICOS) which function as immune checkpoint and belong to the CD28-superfamily, CD154 (CD40 ligand) which is a TNF superfamily molecule binding to CD40 on antigen-presenting cells, CD69 which is a C-type lectin expressed on activated T- and NK-cells, and the MHC class II surface receptor HLA-DR (on CD4+ and CD8+ T-cells).

Thus, even though B-cells readily interact with other cells of the immune system, we observed no significant changes in numbers of T-cells or NK-cells, or in T-cell activation parameters, after the B-cell depleting intervention, and this is in line with observations after rituximab treatment in RA patients [47].

Reduced NK-cell function in ME/CFS patients has been reported in several studies [7,48,49]. In the present study, no NK-cell functional tests were performed. Only investigating quantitative aspects of lymphocyte subsets during follow-up, with no functional assays (e.g. for NK-cells) represents a limitation in the present study. Thus, we cannot exclude functional deficits in specific lymphocyte subsets at baseline or after intervention. We have investigated rather limited subgroups of T-lymphocyte subsets during follow-up. Therefore, other T-lymphocyte subpopulations not investigated in the current study may vary during rituximab treatment.

Studies of B-cell subsets in patients with ME/CFS have not revealed consistent and reproducible differences from healthy controls. A recent study reported increase in frequency and expression of CD24 on B-cells confined to IgD+ subsets, compared to healthy controls [50]. One study on B-lymphocyte subsets in ME/CFS patients showed a higher percentage of naïve and transitional B-cell subsets compared to healthy controls [9]. In contrast, another study showed decreased naïve B-cells and increase in memory B-cells [51]. Another study reported no B-cell abnormalities, but T-cell and NK-cell abnormalities including increased levels of T-regulatory cells associated with ME/CFS versus healthy [52]. T- and NK-cell abnormalities were also reported when comparing ME/CFS patients with moderate or severe disease versus healthy controls [53].

The changes in serum immunoglobulin levels, from baseline to 24 months follow-up, after six rituximab infusions in most patients, were included to assess possible toxicity from hypogammaglobulinemia after rituximab maintenance treatment. Through 24 months follow-up, there were slight but significant decreases in IgG, IgA and IgM. The IgG immunoglobulin levels at 24-months were still well within the normal ranges, while the drop in IgM levels were more pronounced, some patients having levels decreased to below the normal range at 24-months. Should one extend the B-cell depletion periods further, a subgroup of patients will be expected to develop hypogammaglobulinemia with clinical risk for infections. However, selective preservation of specific antibodies (or autoantibodies), and reductions of others, may occur depending on whether the actual plasma cells express the CD20 antigen or not, as shown in a mouse model of RA demonstrating that rituximab specifically depleted short-lived autoreactive plasma cells [54].

Comparing baseline parameters between patients achieving clinical response after B-cell depletion or no response, we observed a significantly higher mean serum IgG level in non-responders compared to responders. A higher IgG level could reflect a nonspecific polyclonal B-cell activation. Previous reports in autoimmune diseases, especially RA, on associations between baseline immunoglobulin levels and subsequent response to B-cell depletion therapy vary. A study among RA patients having relapsed following prior disease-modifying anti-rheumatic drugs or TNF antagonists, reported higher IgG levels to be associated with non-response after rituximab therapy [55]. Contrary, serum IgG levels were above the upper normal value in a higher fraction of patients with refractory RA responding to rituximab treatment than among non-responders [56]. A review article discussing predictive factors in RA, suggested that lower serum levels of interferon-γ and of BAFF, presence of the Fcγ receptor III genotype, the C/G-174 IL-6 polymorphism, or rheumatoid factor positivity, were associated with clinical response after rituximab therapy [57].

These possible association between low IgG level and clinical response after B-cell depletion therapy in ME/CFS reported here is uncertain due to the low number of patients, and will be analyzed after un-blinding of the ongoing randomized, double-blind phase-III study in Norway, with rituximab maintenance or placebo.

The data presented in this study cannot lend support to any specific disease mechanism. The clinical data from the previous trials evaluating B-cell depletion for ME/CFS [11,12], indicates that a subgroup of ME/CFS patients has an immunological disease in which B-cells and possibly antibodies may be important for symptom maintenance. However, the present data neither strengthen nor weaken this hypothesis. Taken together, the data reported here do not support B-cell induced quantitative modifications of specific T-lymphocyte subsets or NK-cells as mechanisms for the observed clinical responses to B-cell depletion therapy in ME/CFS. Non-responders to rituximab treatment had higher baseline levels if serum IgG than responders. ME/CFS patients had increased baseline serum BAFF levels compared to healthy controls, which supports the presence of a chronically activated B-lymphocyte system.
